# Isolation, Purification and Molecular Mechanism of a Peanut Protein-Derived ACE-Inhibitory Peptide

**DOI:** 10.1371/journal.pone.0111188

**Published:** 2014-10-27

**Authors:** Aimin Shi, Hongzhi Liu, Li Liu, Hui Hu, Qiang Wang, Benu Adhikari

**Affiliations:** 1 Institute of Agro-Products Processing Science and Technology, Chinese Academy of Agricultural Sciences/Key Laboratory of Agro-Products Processing, Ministry of Agriculture, Beijing, China; 2 School of Applied Sciences, RMIT University, City Campus, Melbourne, Australia; National Research Council of Italy, Italy

## Abstract

Although a number of bioactive peptides are capable of angiotensin I-converting enzyme (ACE) inhibitory effects, little is known regarding the mechanism of peanut peptides using molecular simulation. The aim of this study was to obtain ACE inhibiting peptide from peanut protein and provide insight on the molecular mechanism of its ACE inhibiting action. Peanut peptides having ACE inhibitory activity were isolated through enzymatic hydrolysis and ultrafiltration. Further chromatographic fractionation was conducted to isolate a more potent peanut peptide and its antihypertensive activity was analyzed through *in vitro* ACE inhibitory tests and *in vivo* animal experiments. MALDI-TOF/TOF-MS was used to identify its amino acid sequence. Mechanism of ACE inhibition of P8 was analyzed using molecular docking and molecular dynamics simulation. A peanut peptide (P8) having Lys-Leu-Tyr-Met-Arg-Pro amino acid sequence was obtained which had the highest ACE inhibiting activity of 85.77% (half maximal inhibitory concentration (IC_50_): 0.0052 mg/ml). This peanut peptide is a competitive inhibitor and show significant short term (12 h) and long term (28 days) antihypertensive activity. Dynamic tests illustrated that P8 can be successfully docked into the active pocket of ACE and can be combined with several amino acid residues. Hydrogen bond, electrostatic bond and Pi-bond were found to be the three main interaction contributing to the structural stability of ACE-peptide complex. In addition, zinc atom could form metal-carboxylic coordination bond with Tyr, Met residues of P8, resulting into its high ACE inhibiting activity. Our finding indicated that the peanut peptide (P8) having a Lys-Leu-Tyr-Met-Arg-Pro amino acid sequence can be a promising candidate for functional foods and prescription drug aimed at control of hypertension.

## Introduction

Peanut (*Arachis hypogaea*) is widely planted, harvested and consumed crop around the world. As oil and protein are major constituents in peanut, it is also a valuable food material in developing countries for managing malnutrition [1]. The oil extracted peanut meal is still nutritionally valuable due to its high protein content (47–55%). However, it is currently underutilized due to its inferior functional properties [2], [3]. Nevertheless, peanut proteins and peptides are known for high level of L-arginine, anti-atherogenic in nature and low anti-nutritional factors [4]–[6]. Because of these reasons, studies on isolation and characterization of peptides from peanut are drawing attention.

Hypertension is closely related to coronary heart disease and stroke and it is one of the top three medical conditions causing death. An effective treatment of hypertension also reduces the risk of other diseases such as myocardial infarction and end-stage renal disease [7]. Among the existing treatments, angiotensin I-converting enzyme (ACE, EC 3.4.15.1) inhibitors help in regulating blood pressure through reducing the generation of Angiotensin II, which can strongly shrink vessels and enhance the blood pressure [8]. Hence, a more effective inhibition of this enzyme greatly reduces the production of angiotensin and ultimately reduces the risk of hypertension [9]. At present, a number of synthetic inhibitors such as captopril and enalapril have been developed as antihypertensive drugs [10], [11]. However, these antihypertensive drugs cause side effects including skin rashes, cough, and other disturbances, which inevitably restrict their application [12].

Bioactive peptides usually contain 3–20 amino acid residues per molecule [13]. A number of research reports have shown that bioactive peptides are capable of imparting antioxidative [14], antimicrobial [15], immunomodulatory [16], and antihypertensive effects [17]. The ACE inhibiting activity of bioactive peptides has also been reported by Pihlanto-Leppälä [18]. Li et al. produced ten peptides from buckwheat with ACE inhibiting activities (IC_50_) ranging from 4 µM to 100 µM using pepsin, chymotrypsin and trypsin [19]. A number of methods can be used to produce bioactive peptides with different amino acid sequences [13].

From literature, most of researchers have worked only on the ACE inhibitory ability of peptides [20]–[22]. There are only a limited number of studies dealing with the interaction between peptide and ACE [4], [23]–[25]. Although crystal structure of ACE inhibiting peptide compounds and the active sites on ACE have been identified and uploaded to Protein Data Bank [26], [27], trying to find natural antihypertensive agents from different species and investigate their structure-activity relationship still attracts lots of researchers’ interests.

Molecular simulation is a useful tool to analyze the conformational transformation of a small molecule and structure-function relationship of a macromolecule [28], [29]. A number of studies have been done through molecular simulation to elucidate the interaction between small molecules and proteins with a high degree of accuracy [30], [31]. It appears that no study has been undertaken to elucidate the ACE inhibitory mechanism of peanut peptides using molecular simulation.

In this context, we isolated ACE inhibiting peptides from peanut protein isolate (PPI) and examined their ACE inhibitory activity. The amino acid sequence of a particular peanut peptide (P8) which showed the highest ACE inhibitory activity was determined. The mechanism of ACE inhibition of this particular peptide (P8) was investigated through molecular simulation including molecular dynamics and molecular docking.

## Materials and Methods

### Materials

PPI was purchased from Lanshan Group (Shandong, China). The following commercial enzymes were obtained: Alcalase (protease)-(Novo Industri A/S, Bagsvaerd, Denmark); N120P (protease)-(Kerry Co., Tralee, Ireland) and ACE (protease from rabbit lung)-(Sigma Chemical Co., St. Louis, MO, USA). Hippuryl-histidyl-leucine (HHL) was purchased from Sigma Chemical Co. (St. Louis, MO, USA). Methyl Cyanide (Merck & Co., Inc., Rahway, NJ, USA) and trifluoroacetic acid (TFA) (Fluka Co., Buchs, Switzerland) were purchased and were of chromatographic grade. All the other materials were of analytical grade and were used as received.

### Preparation and separation of peanut peptides

The peanut peptide samples were produced using an enzymatic hydrolysis method as reported by Wang et al. [32]. Specifically, 4% (w/w) PPI solution was stirred and pre-heated at 80°C for 10 min and then the temperature was reduced to 53°C. After adjusting to pH 8.0 using NaOH (1 M), 3637 U/g alcalase (protease) was added to the PPI solution and the mixture was further stirred for 105 min while maintaining the temperature at 53°C. Subsequently, the mixture was adjusted to pH 6.0 (using 1 M NaOH) and a temperature of 57°C. Finally, 2061 U/g N120P (protease) was added into the solution and hydrolysis was carried out for further 65 min. After completion of hydrolysis, the solution was heated at 90°C using a water bath (CS501-SP, SiDa Science Instruments Inc., China) for 10 min to deactivate the enzymes. The peanut peptides were obtained from the supernatant after centrifugation at 1503 g for 15 min.

The peptide molecules having molecular weight below 1 kDa were obtained through ultra-filtration (filter membranes, MWCO: 1 kDa). The filtered peptide mass was freeze dried at –50°C for 20 h (freeze dryer, LGJ-25, Sihuan, China). This freeze dried peanut peptide powder was sealed in zip-lock bags and stored in a humidity and temperature controlled chamber (25°C and 10% relative humidity).

### Determination of ACE inhibiting activity (*in vitro* tests)

ACE inhibiting activity was measured using Cushman and Cheung’s method with slight modification [33], [34]. Specifically, 20 µL of 4-hydroxyethylpiperazine ethane sulfonic acid (HEPEs) buffer solution (pH 8.3) was mixed with 20 µL sample solution in a 0.5 ml centrifuge tube. Then, 15 µL ACE solution was added into this tube and warmed at 37°C for 3 min. The reaction was started by adding 50 µL Hip-His-Leu (HHL) solution and continued for 30 min maintaining the same temperature (37°C). Finally, 50 µL HCl solution (1 M) was added to the fully reacted mixture to terminate the reaction. This reacted mixture was filtered through a micro-filtration membrane (0.45 micron diameter) and the filtrate was used for further analysis.

The analysis of the sample solutions was performed by HPLC (Breeze, Waters Co. Milford, MA), consisting of a pump (Waters 2795) and a UV detector (Waters 2487). SunFireTM-C18 (4.6×150 mm, 5 µm, Waters Co. Milford, MA, USA) chromatographic column was used and was operated at 30°C. An injection volume of 20 µL was used at a flow rate of 0.4 ml/min. The mobile phase consisted of methyl cyanide, water and TFA at volume ratio of 50∶50∶0.05. The wavelength used for detection was 228 nm.

The ACE inhibiting activity (%) was expressed in terms of hippuric acid content and was calculated using (1).
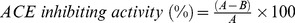
(1)where, A(mAu*s) is the area under the curve of hippuric acid in blank sample; B (mAu*s) is the area under the curve of hippuric acid in test sample. And IC_50_ represents the concentration of an ACE inhibitor needed to inhibit 50% of the ACE activity.

### Analysis of ACE inhibition kinetics of peanut peptide

The ACE inhibition kinetics of peanut peptide was investigated according to the previous method [35]. Several samples with different peanut peptide concentration (0 mg/ml, 0.1 mg/ml and 0.5 mg/ml) were prepared, and under different concentration of substrate, the inhibitory reaction ratio was determined and the ACE inhibition kinetics could illustrated.

### Identification of antihypertensive activity (*in vivo* tests)

The *in vivo* tests for antihypertensive activity of peanut peptide was conducted using a previous method [36], and whole tests were conducted in Chinese Medicine Experiment Center of Hubei College of Traditional Chinese Medicine, authorized by SATCM (State Administration of Traditional Chinese Medicine, China). The male spontaneously hypertensive rats (SHRs) was used for the experimental model while the average weight of each rat was between 210 and 240 g and four rats were numbered as one group for feeding. These rats had free access to food and drink in their cages located in a 25°C room temperature with 12 h of light cycle. After 1 week for adaptation to this environment, the determination of blood pressure was conducted and the comparison of different group was investigated. The SHRs were randomly divided into five groups, named blank control group, low-dose group (100 mg/kg body weight (BW)), intermediate-dose group (500 mg/kg BW), high-dose group (1000 mg/kg BW) and drug control group (40 mg Captopril/kg BW).

Short-term antihypertensive activity: Peanut peptide or captopril was dissolved in 1 ml normal saline according to the dosage of each group while blank control group only contained a gavage of normal saline with same volume. All the SHRs were kept at 37°C for 10 min and then the gavage was conducted. The systolic blood pressure (SBP) of each SHRs was determined using noninvasive tail arterial blood pressure measurement analysis system (ZH-HX-Z, ZhengHua Biological Instrument equipment co., LTD, China) at 0, 1, 3, 5, 7, 9 and 12 h after the gavage. Each SHR was measured for three times.

Long-term antihypertensive activity: Through the same method of sample preparation, these SHRs were feeded at the same time (9∶00 am–10∶00 am) of each day and the feeding continued for 28 days. And the determination of SBP was conducted after 0, 7, 14, 21 and 28 days using noninvasive tail arterial blood pressure measurement analysis system (ZH-HX-Z, ZhengHua Biological Instrument equipment co., LTD, China). Each SHR was measured for three times.

### Reversed-phase chromatography of ACE inhibiting peptides

The dried peanut peptide powder having molecule weight below 1 kDa was first dissolved in water to make a peptide solution with 100 mg/ml concentration. Then this solution was fractionated using a Sephadex G-15 column (16×600 mm). The temperature, injection volume and flow rate of the solution were maintained at 30°C, 3 ml and 0.3 ml/min, respectively. Each fraction was collected for 10 min and the entire process was repeated four times. Finally, fractions having particular (same) elution time were collected and freeze dried. The IC_50_ of each fraction was determined as *in vitro* tests.

The fraction with the lowest IC_50_ was further fractionated (purified) by a semi-preparative reversed-phase high performance liquid chromatography (RP-HPLC) (ProStar 218, Varian Instrument Group, Sugar Land, TX), with Varian C18 (21.2×150 mm) column. The temperature, injection volume of the sample and flow rate of mobile phase were 25°C, 1 ml and 10 ml/min, respectively. The mobile phase was composed of solution A (TFA/water: 0.05/100, w/w) and solution B (TFA/methyl cyanide: 0.05/100, w/w). For elution times of up to 50 min, concentration of solution A reduced from 95% (w/w) to 70% (w/w) while the concentration of solution B increased from 5% to 30%, respectively. The detection wavelength used in these tests was 214 nm. The purified peptide faction was freeze dried and stored in a controlled humidity chamber (25°C and 10% relative humidity) for further analysis.

The composition or relative purity of each fraction was examined using HPLC (Breeze, Waters Co. Milford, MA) which consisted of a C18 column, a pump (Waters 2795) and a UV detector (Waters 2487). Twenty microliter sample was injected each time and the sample temperature and the detection wavelength were 30°C and 214 nm, respectively. The elution conditions used in this test was similar to those presented above.

### Determination of peanut peptide sequence

One microliter (1 µl) of sample solution was mixed with 1 µl matrix solution composed of α-cyano-4-hydroxy-transcinnamic acid (Sigma) (5 mg/ml, w/v), TFA (0.1%, w/w) and methyl cyanide (50%, w/w). The above blended solution was transferred onto the MALDI target plates and analyzed using an ABI 4700 Proteomics Analyzer MALDI-TOF/TOF-MS (Applied Biosystems, Foster City, CA) [37]. The test was conducted under positive ion mode and the frequency, acceleration voltage and wavelength of the laser (source) were 20 Hz, 20 kV, and 355 nm, respectively. Scanning range for the peptide mass finger printing (PMF) of matrix and sample was 600–1000 Da. The standard fibrino-peptide B was used as the external standard. After an initial MS scanning, MS/MS analyses were performed. The amino acid sequence of peanut peptide was obtained by using DeNovo Explorer (Applied Biosystems, Foster City, CA).

### Molecular simulation of ACE-peptide interaction

Molecular simulations were conducted using Discovery Studio software package (DS 3.5) (Accelrys Inc., San Diego, CA). The structure of peanut peptide was constructed on the basis of amino acid sequence was determined in material and method section. The native crystal structure of ACE was obtained from Protein Data Bank (protein entry 1O8A; resolution at 2.00 Å).

Molecular docking simulation was executed to accurately predict the docking of ligands into active sites of protein. For this purpose, CDOCKER module available in DS 3.5 software package was used. A two-step protocol was applied to quantify the receptor-ligand interactions: (1) Docking: attempt was made to dock a ligand into a user defined binding site and (2) Scoring: score values were calculated using suitable scoring functions for each pose (conformation) of the ligands [38], [39]. Initially a protein (1O8A) was prepared by removing all water molecules and CHARMm force field was applied using receptor-ligand interactions tool available in DS 3.5. After the preparation of protein, the active sites on the receptor (ACE) were defined. DS 3.5 defines active sites in three ways; firstly, based on the active cavities of receptor; secondly, based on the volume occupied by known and already existing ligand pose; and thirdly, based on the active site selected manually. We adopted the first definition and the docking sites were determined using the DS 3.5 software. During the docking process, top 10 conformations were generated for each ligand based on docking score. The conformation with the highest -CDOCK energy (score) was then selected for further analysis. Interaction energy of the ligand-receptor interaction was calculated and interaction energy was illustrated in 2D and 3D images.

Molecular dynamics simulation was performed in the DS 3.5 simulation package with CHARMm force field. Specifically, ACE-peptide complex was first pretreated in the absence of water molecule and later a 7 Å solvation shell was added. At same time, 0.145 M NaCl was used to simulate the human environment. Two minimization cycles (steepest descent and conjugate gradient) were performed until the RMS of energy gradient was <0.1 kcal/mol·Å [40]. The steepest descent cycle was performed with 2000 steps (time step: 0.001 ps) while conjugate gradient was performed with 1000 steps (time step: 0.001 ps). The SHAKE algorithm was applied throughout the MD simulation to hold all the bonds involving hydrogen atoms. The long-range electrostatic forces were treated with PME method. After minimization, the sample was gradually heated to a target temperature from 50 to 300 K over an interval of 5 ps. After this heating process, a 5000 steps long (time step: 0.002 ps) equilibration phase was applied. The production stage was performed in 50000 steps using a time step of 0.002 ps using NPT canonical assembly. The decay time for the temperature coupling was 5.0 ps.

### Statistical analysis

Duncan’s multiple comparison method was used to determine the significant difference between mean values. A confidence level was set at *p<0.05* and the SAS software (SAS Institute Inc., Cary, NC, USA) was used in these statistical analyses.

Student’s t-test was used for statistical analysis of the difference in SBPs before and after peptide administration (*p<0.01*) (Daniel 1987). Data are expressed as means and standard errors and data point with different number of dark spot showed significant different in line chart.

## Results and Discussions

### Identification and purification of ACE inhibiting peanut peptides

**Figure 1:**
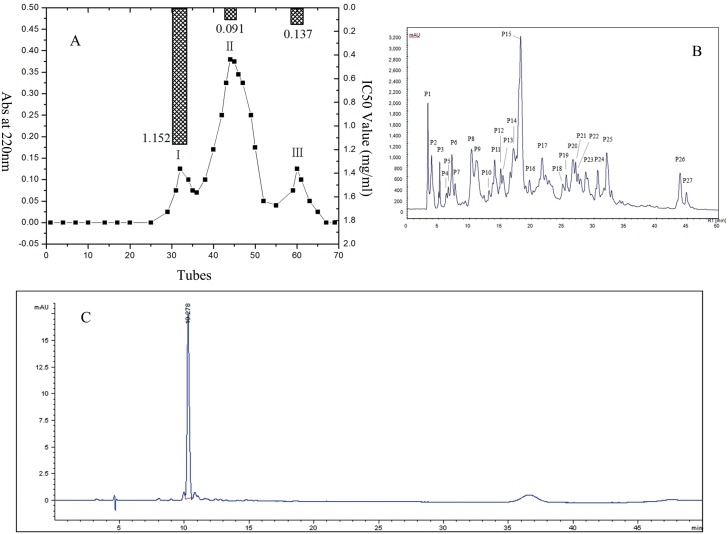
Composition and ACE inhibiting peanut peptides. A: separation chromatogram of peanut peptides; B: composition of PP-II; C: composition (relative purity) of P8 in PP-II.

Fig. 1A shows that there are three main fractions in peanut peptide having molecule weight below 1 kDa, namely PP-I, PP-II and PP-III. The IC_50_ values of these three fractions were determined and found to be 1.152 mg/ml, 0.091 mg/ml and 0.137 mg/ml, respectively. It has been reported that the hydrolysates produced by alcalase from food proteins showed potent antioxidant activity [41] and ACE inhibiting activities [42], [43]. These bioactivities primarily result from the endo-peptidase properties of various bioactive peptides produced by alcalase. The ACE inhibiting activity observed in the peptides obtained from PPI can be attributed to hydrolysis of PPI by alcalase. Therefore, peanut peptides were isolated from hydrolysates produced by alcalase and three peptide fractions having different ACE inhibiting activity were obtained. Out of these peptide fractions, PP-II exhibited the highest ACE inhibiting activity; hence, it was chosen for further investigation.

The RP-HPLC profile of PP-II is presented in Fig. 1B. As can be seen from this figure, there are 27 components in PP-II. As shown in Table 1, the ACE inhibiting activity of component P8 was the highest (85.77%). Because of its highest ACE-inhibitory activity, this component (P8) was chosen for further purification and structure analysis.

**Table 1:** The ACE inhibiting ratio of each HPLC fraction in peanut peptide (PP-II)*.

*Fraction*	*Inhibition (%)*	*Fraction*	*Inhibition (%)*
P1	66.20	P15	24.19
P2	47.84	P16	60.67
P3	66.79	P17	64.33
P4	57.64	P18	49.19
P5	24.29	P19	60.72
P6	60.36	P20	57.21
P7	30.05	P21	59.35
P8	85.77	P22	48.25
P9	23.47	P23	52.79
P10	54.27	P24	13.09
P11	66.15	P25	40.1
P12	55.79	P26	54.07
P13	60.89	P27	53.85
P14	55.08		

*Peanut peptide concentration is 100 µg/ml.

Fig. 1C shows the composition or relative purity of the component P8. As only a single peak is observed, it indicates that P8 is composed of peanut peptide with same molecular weight.

### Analysis of ACE inhibitory kinetic and in vivo antihypertensive activity

**Figure 2:**
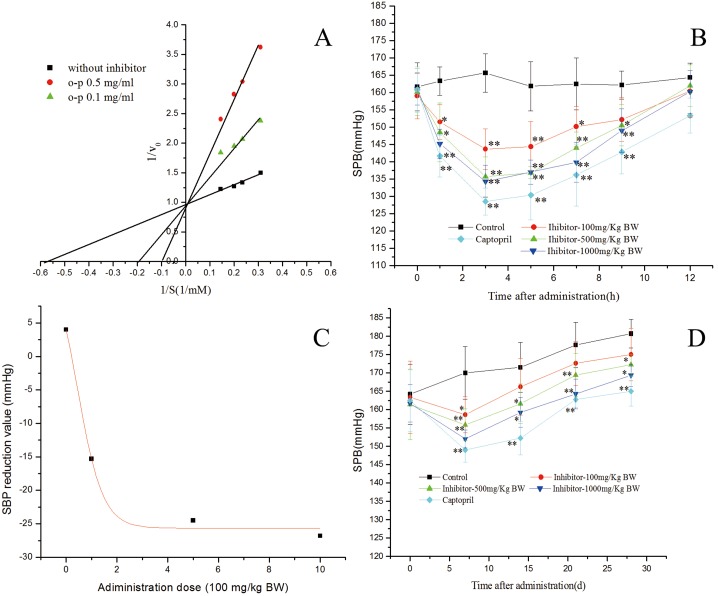
In vivo anti-hypertensive activity and action kinetic of peanut peptide. A represents the effect of one time dose of peanut peptide on SBP in the SHR; B represents the Sigmidal model of peanut peptide dose effect; C represents the effect of multiple dose of peanut peptide on SBP in the SHR; D represents Lineweaver-Burk model of ACE inhibition of peanut peptide.

Fig. 2 (A) shows the mode of inhibition of the ACE-catalyzed hydrolysis of Hip-Leu-His and from the Lineweaver-Burk curve, it is competitive inhibition type [44]. From literature, many researchers have reported the non-competitive ACE inhibitory peptides such as Leu-Ile-Tyr, Tyr-Leu-Tyr-Glu-Ile-Ala-Arg from human serum trypsin hydrolysate [45], but most ACE inhibitors either from snake venom [46] or derived from food protein hydrolysates [47] belong to the competitive mode. And the competitive inhibitors are able to enter the ACE protein molecule, interact with the active sites and prevent substrate binding, which could benefit the application of peanut peptide as a potential antihypertension medication.

The *in vivo* antihypertension experiments of peanut peptide has been conducted and the results are illustrated in Fig. 2. As we can see from panel B, a single administration of peanut peptide at doses of 100, 500 and 1000 mg/kg BW can significantly influence the systolic blood pressure (SBP) in SHRs. Comparing with captopril, the antihypertension activity of peanut peptide shows same trends [48], [49]. Specifically, at 3 h after the gavage of the peanut peptide, the SBP shows the lowest value (145 mmHg at 100 mg/kg BW, 136 mmHg at 500 mg/kg BW and 134 mmHg at 1000 mg/kg BW) and the total decline is over 20 mmHg. In addition, the antihypertensive results of control group, low-dose group (100 mg/kg BW), intermediate-dose group (500 mg/kg BW) and high-dose group (1000 mg/kg BW) was analyzed in Fig. 2 (panel C). With the enhancement of peptide doses, the SBP of SHRs trends to be stable. This results illustrate that the peanut peptide may be a safe antihypertensive agent which will not reduce blood pressure excessively.

Thereafter, the effect of long-term administration of peanut peptide at the doses of 0, 100, 500 and 1000 mg/kg BW was examined in SHRs, which showed SBP over 160 mmHg. As we can see from Fig. 2 (panel D), peanut peptide at the dose of 100, 500 and 1000 mg/kg BW can produce significant reduction in SBP of SHRs after one week of administration, although the SBP of SHRs shows a certain of increasing from the second week. In addition, with the increase of gavage dose, this antihypertensive activity is also enhanced and total trend is similar to the captopril [50]. Though the captopril show stronger antihypertensive activity during the long term experiment, the potential of peanut peptide as the antihypertensive agent is still worth to be investigated because of its plant protein source.

### Structural analysis of ACE inhibiting peptides

The structural analysis of peanut peptide (P8) was conducted using MALDI-TOF/TOF-MS in combination with the DeNovo Explorer and the outcome is listed in Table 2. Specifically, the amino acid sequence of P8 is Lys-Leu-Tyr-Met-Arg-Pro and the molecular weight is 808.8 Da, which is close to the theoretical value (807.7 Da). The IC_50_ value of P8 is 0.0052 mg/mL (6.42 µM) (Table 2), which is highest among the reported ACE inhibiting activity values of peanut peptides [4], [25].

**Table 2:** Composition and basic properties of peanut peptide (P8).

*Peanut peptide (P8)*	*Results*
Amino acid sequence	Lys-Leu-Tyr-Met-Arg-Pro
Molecular weight (theoretical)	807.7
Molecular weight (experimental)	808.8
IC_50_	0.0052 mg/mL (6.42 µM)

It is reported that the ACE inhibiting peptides usually contain 2 to 12 amino acids and their molecular weights generally range from 150 to 800 Da [51]. Guang and Phillips produced a peanut tetrapeptide (Lys-Ala-Phe-Arg) with ACE inhibiting activity of 16.9 µg/ml [52]. Li et al. reported the preparation of an ACE inhibiting hexapeptide (Val-Thr-Pro-Ala-Leu-Arg) with IC_50_ value of 82.4 µM) from mung bean protein isolate using alcalase [43]. It is reported that ACE inhibiting peptides usually contain Pro, Lys or aromatic amino acid residues and our data also confirm this observation.

### Molecular docking of peanut peptide and ACE

Table 3 shows the docking score of top 10 best structural configurations (poses). Specifically, the -CDOCKER energy and -CDOCKER interaction energy for the best pose are 135.306 kcal/mol and 134.928 kcal/mol and both values indicate to a strong interaction between ACE and the peanut peptide. The best pose between an active site of ACE and a peptide is achieved at the highest interaction energy value.

**Table 3:** Pose number, Docking score and interaction energy of top 10 best poses.

*Pose number*	*-CDocker Energy*	*-CDocker Interaction Energy*	*Interaction energy (kcal/mol)*
1	135.306	134.928	−393.362
2	111.015	107.755	−187.615
3	101.236	103.146	−15.006
4	85.2762	103.316	−247.644
5	81.1103	88.0692	−35.021
6	81.1103	88.0692	−35.021
7	81.1103	88.0692	−35.021
8	81.1103	88.0692	−35.021
9	81.1103	88.0692	−35.021
10	81.1103	88.0692	−35.021

**Figure 3:**
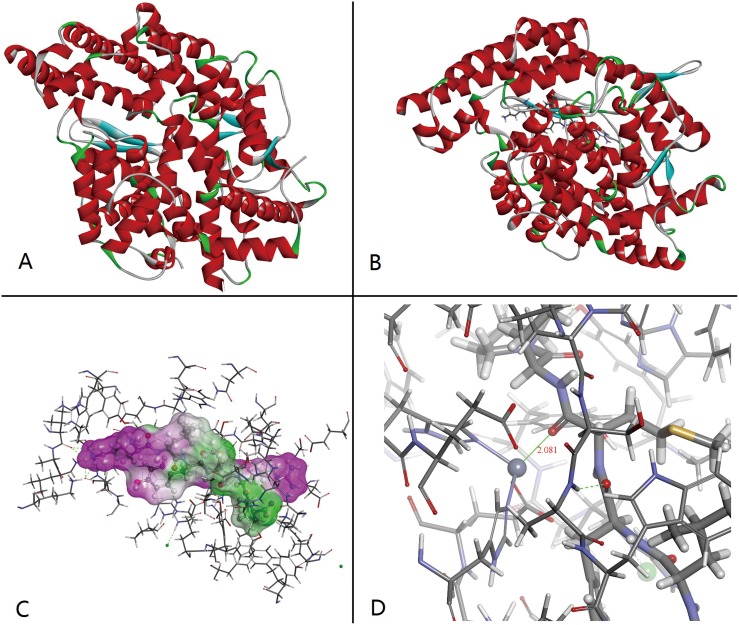
3D images obtained from native ACE (PDB 1O8A) (Panel A) and molecular docking simulation (Panel B: ACE-peptide complex; Panel C: interaction between peptide and ACE residues; Panel D: interaction between peptide and zinc atom).

Fig. 3 presents the best interaction pose obtained through molecular docking simulation. Panel A shows the structure of ACE. As can be seen from panel B and panel C, the docking was successful and the ligand was docked in the active site of ACE [53]. Panel B shows the key residues located at ACE and peanut peptide interface primarily contribute to the interaction energy (Table 3). Panel B also illustrates that active pocket of ACE was occupied by peanut peptide, which could explain the ACE inhibiting activity of peanut peptide (P8) [54]. Besides, the interaction between Zn^+^ and ligand also shows greater influence on the ACE inhibiting activity [51] and a shorter distance or closer proximity is necessary for creating such interaction. For example, in ACE-enalapril complexation (PDB entry: 1UZE), the distance between Zn^+^ and carboxyl groups on enalapril is 2.016 Å due to which metal-carbonyl moiety can easily be formed. From panel C (Fig. 3), the distance between Zn^+^ and carboxyl group on P8 is 2.018 Å, which also favors the formation of metal carbonyl.

**Figure 4:**
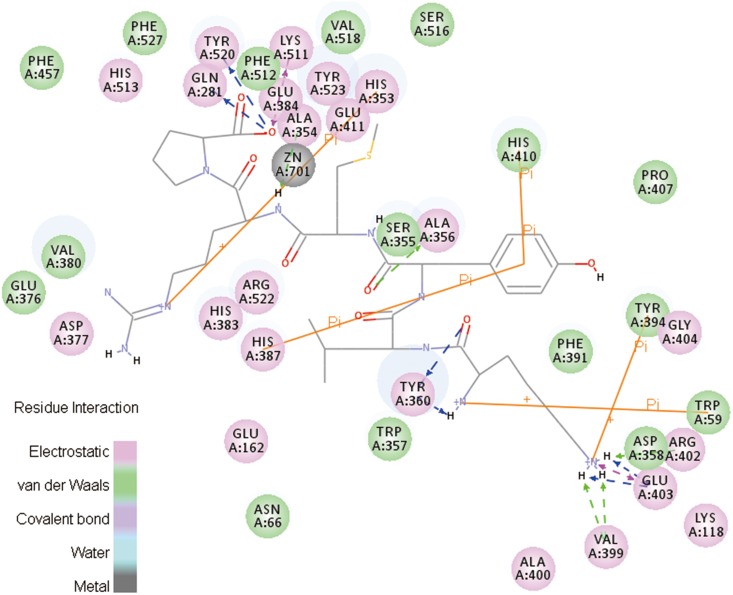
2D diagram showing interactions between peptide and ACE amino acid residue obtained from molecular docking simulation.

The specific interaction between ligand (P8) and receptor (ACE) is illustrated in Fig. 4. Hydrogen, electrostatic and Pi bonds are the three main kinds of bonds involved in this interaction. Specifically, hydrogen bonds are formed at Glu:403, Arg:402, Val:399, Tyr:360, Ala:356, Ala:354, Tyr:520, Gln:281 of ACE while electrostatic bonds are formed act at Glu:403 and Lys:511. In addition, Pi-bonds are formed at Trp:59, Typ:394, His:387, His:410 and His:353.

Tyrosine (TYR) is an aromatic amino acid. Its carboxyl functional group can be a hydrogen acceptor while Ar-H or Ar-OH can be good hydrogen donors. Ala and Val are aliphatic amino acids which favor formation of hydrogen bond as well as pendant group (hydrogen donor) at their carboxyl groups. Glutamic (Glu) is an acidic amino acid with two carboxyl groups which generates hydrogen bonds easily [55]. For Gln, the hydrogen atoms in amine groups greatly favor the formation of hydrogen bonds. Arginine (Arg) is a zwitterionic amino acid capable of forming hydrogen bond either as donor or receptor of hydrogen atom (H) [56]. Electrostatic attractive force can be easily generated between Glu and amino group on P8 due to negative charge of Glu. LYS is a basic amino acid and the positively charged amino group can attribute to the formation of electrostatic bond. The benzene ring in Trp, Typ and His favors the formation of Pi bonds.

### Molecular dynamics simulation of peanut peptide and ACE

The molecular dynamics simulation (Table 4) shows that six new interaction bonds (including hydrogen and electrostatic bonds) were formed and two Pi bonds were disappeared. Besides, the bonding distance of all hydrogen bonds was below 2.5 Å, which confirms the successful molecular docking [57]. As a consequence, the interaction energy between peanut peptide and ACE increased from 393.362 to 556.746 kcal/mol, which indicates to a compound structure having quite strong interaction energy.

**Table 4:** Interaction between peptide and ACE amino acid residues before and after molecular dynamics*.

*Before Molecular Dynamics*		*After Molecular Dynamics*	
*Interaction*	*Distance (Å)*	*Interaction*	*Distance (Å)*
A:GLN281:H(E22)-pp:O(120)	2.24543	A:GLN281:H(E22)-pp:O(119)	2.084
A:ALA356:H(N)-pp:O(64)	2.22196	A:HIS353:H(E2)-pp:O(105)	1.95423
A:TYR360:H(H)-pp:O(24)	2.33038	A:HIS353:H(E2)-pp:O(120)	2.14551
A:LYS511:H(Z1)-pp:O(120)	1.88773	A:TYR360:H(H)-pp:O(24)	2.42925
A:TYR520:H(H)-pp:O(120)	2.13421	A:LYS511:H(Z1)-pp:O(119)	1.91908
pp:H(3)-A:TYR360:O(H)	1.91999	A:LYS511:H(Z1)-pp:O(120)	1.75272
pp:H(20)-A:ARG402:O	1.91365	A:TYR520:H(H)-pp:O(120)	2.14714
pp:H(20)-A:GLU403:O(E1)	2.22408	A:ARG522:H(H11)-pp:O(43)	1.75486
pp:H(21)-A:VAL399:O	2.28661	A:TYR523:H(H)-pp:O(81)	2.32959
pp:H(22)-A:VAL399:O	2.46477	pp:H(2)-A:TYR360:O(H)	1.68598
pp:H(22)-A:GLU403:O(E1)	2.47199	pp:H(21)-A:GLU403:O(E2)	1.83082
pp:H(83)-A:ALA354:O	2.1418	pp:H(22)-A:ALA400:O	2.11338
pp-A:HIS410	4.01477	pp:H(22)-A:GLU403:O(E1)	1.8652
pp-A:HIS387	5.25981	pp:H(22)-A:GLU403:O(E2)	2.46272
A:TRP59-pp:N(1)	6.13909	pp:H(62)-A:ARG402:O	2.28798
A:TYR394-pp:N(19)	6.63104	pp:H(83)-A:HIS353:O	2.18907
A:HIS353-pp:N(95)	5.08307	pp:H(99)-A:GLU162:O(E2)	1.72534
		pp:H(102)-A:GLU376:O(E1)	1.94308
		pp-A:HIS410	3.819
		pp-A:HIS410:N(E2)	3.80281
		HIS353-L:pp:N(95)	5.09191
		TRP357-L:pp:N(1)	6.26867

*A is the abbreviation of ACE and pp represents peanut peptide. In A:XXX123:Y(00), A represents ACE, XXX represents the abbreviation of amino acid molecule, 123 represents the serial number of this amino acid molecule on ACE, Y represents the atom involved into the interaction and 00 represents the serial number of this atom on this amino acid molecule. In, pp:X(123), pp represents peanut petides, X represents the atom involving into the interaction and 123 represents the serial number of this atom on the peanut peptide.

**Figure 5:**
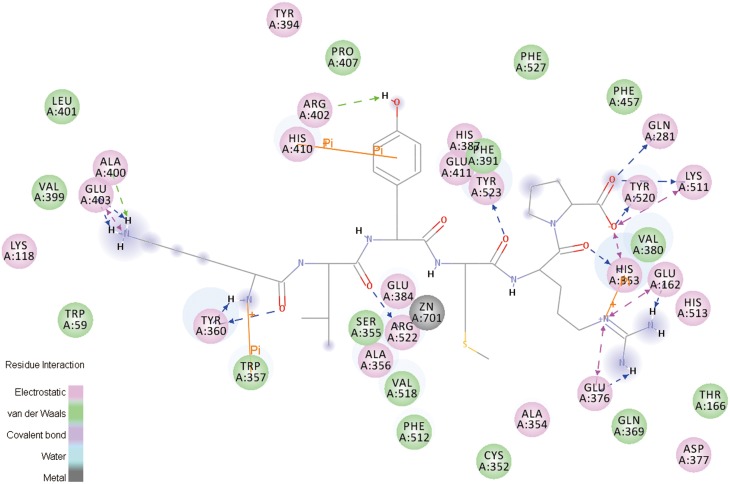
2D diagram showing interactions between peptide and ACE amino acid residues obtained from molecular dynamics simulation.

As can be seen from Fig. 5, all interactions including hydrogen bonds, electrostatic bonds and Pi bonds are specifically marked. Hydrogen bonds are formed at Gln:281, His:353, Tyr:360, Tyr:520, Tyr:523, Arg:402, Arg:522, Glu:162, Glu:376, Glu:403, Ala:400 and Lys:511 of ACE while electrostatic bonds are formed at Glu:403, Lys:511, His:353, Glu:162 and Glu:376. Similarly, Pi-bonds are formed at His:353, His:410 and His:357. Similar to the data shown in Fig. 4, Glu, Arg, Tyr and Ala are still the amino acid residues favoring the formation of hydrogen bonds. Three new amino acid residues including His:353, Glu:162 and Glu:376 contribute the formation of electrostatic bonds. These observations indicate that, although the number of Pi bonds decreases, the interaction between peptide and ACE becomes much stronger explaining the robust structural stability of ACE-peptide compound. The ACE inhibitory activity of peanut peptides can also be attributed to this strong interaction between ACE and peanut peptides.

**Figure 6:**
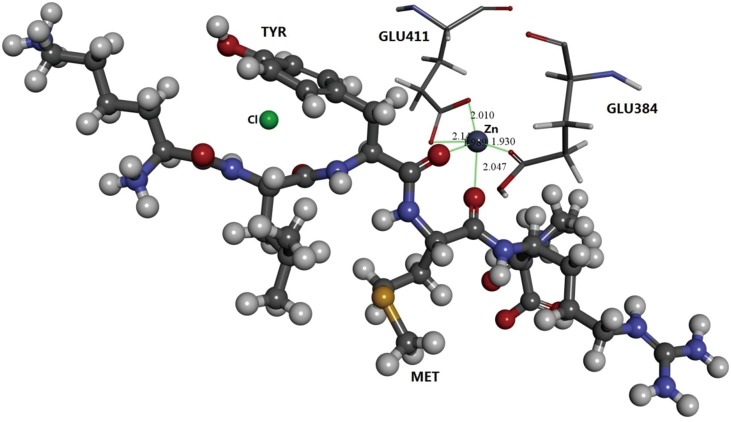
Schematic diagram showing interaction of peanut peptide and ACE amino acids around a zinc atom. The stick-ball model represents peanut peptide; thin stick model represents ACE amnio acide residues; red ball and red stick represent oxygen; blue ball and blue stick represent nitrogen; black ball and black stick represent carbon; gray ball and gray stick represent hydrogen; yellow ball represents sulphur.

The interaction between zinc atom and peanut peptide in ACE-peptide compound is shown in Fig. 6. As shown, the zinc atom is surrounded by Glu:411, Glu:384 from ACE and Tyr, Met from peanut peptide. The distance between zinc atom and carboxyl group of these amino acid residues ranges from 1.989 to 2.140 Å. As reported by Natesh et al. (2004), the formation of metal carboxylic coordination bond between zinc atom in ACE and enalapril brings about the observed ACE inhibiting behavior [27]. The distance between Zn^+^ and carboxyl groups in enalapril is 2.016 Å. At the same time, the distance between zinc atom and each of the five neighboring oxygen atoms is shorter than the sum (2.14 Å) of radius of covalent bond, oxygen atom (1.4 Å) and zinc atom (0.74 Å) [58]. Thus, as shown in Fig. 6, the zinc atom and carboxyl groups are capable of forming five metal carboxylic coordination bonds and are responsible for the structural stability of ACE-peptide compound. This stability is also responsible for imparting considerable ACE inhibiting potential.

## Conclusion

Peanut peptides having ACE inhibitory activity were successfully produced from PPI through enzymatic hydrolysis. Peanut peptide fraction (P8) having the highest ACE inhibiting activity (85.77%) was separated and purified through chromatography. The IC_50_ of this fraction (P8) for ACE inhibition was 0.0052 mg/ml (6.42 µM) which is much lower than that of the ACE inhibiting peanut peptides reported so far. The purity of this particular peanut peptide (P8) was verified and its amino acid sequence was determined. P8 was found to be composed of Lys-Leu-Tyr-Met-Arg-Pro amino acid residues in sequence and had the molecular weight of 808.8 Da. This peanut peptide is a competitive ACE inhibitor and it showed significant short term (12 h) and long term (28 days) antihypertensive activity. The molecular docking simulation showed that P8 was successfully docked in the active pocket of ACE with the highest -CDOCKER energy (score) of 135.306 kcal/mol. Hydrogen bonds, electrostatic bonds and Pi bonds were found to contribute to the structural stability of ACE-peptide compound. The molecular dynamics simulation showed that peanut peptide and ACE had very strong interaction due to increased number of hydrogen bonds and the formation of metal carboxylic coordination bond between zinc atom and carboxyl group of peanut peptide. Fourteen hydrogen bonds, five electrostatic bonds and three Pi bonds were found to form between peanut peptide and ACE amino acid residues. Five metal carboxylic coordination bonds were found to form around zinc atom, fully occupying the active sit of ACE. All these interaction forces were found to be responsible for the high ACE inhibiting ability of this peanut peptide (Lys-Leu-Tyr-Met-Arg-Pro).
